# Cell-Free DNA in the Investigation of Miscarriage

**DOI:** 10.3390/jcm9113428

**Published:** 2020-10-26

**Authors:** Emily Colley, Adam J. Devall, Helen Williams, Susan Hamilton, Paul Smith, Neil V. Morgan, Siobhan Quenby, Arri Coomarasamy, Stephanie Allen

**Affiliations:** 1 Tommy’s National Centre for Miscarriage Research, Birmingham Women’s and Children’s Hospital, Birmingham B15 2TG, UK; A.J.Devall@bham.ac.uk (A.J.D.); H.M.Williams.1@bham.ac.uk (H.W.); paul.smith@doctors.org.uk (P.S.); A.Coomarasamy@bham.ac.uk (A.C.); 2 Institute of Metabolism and Systems Research, College of Medical and Dental Sciences, University of Birmingham, Edgbaston, Birmingham B15 2TT, UK; 3 West Midlands Regional Genetics Laboratory, Birmingham Women’s and Children’s Hospital, Birmingham B15 2TG, UK; susan.hamilton15@nhs.net (S.H.); stephanie.allen13@nhs.net (S.A.); 4 Institute of Clinical Sciences, College of Medical and Dental Sciences, University of Birmingham, Edgbaston B15 2TT, UK; 5 Institute of Cardiovascular Sciences, College of Medical and Dental Sciences, University of Birmingham, Edgbaston, Birmingham B15 2TT, UK; N.V.Morgan@bham.ac.uk; 6 Division of Biomedical Sciences, Warwick Medical School, University of Warwick, Coventry CV4 7HL, UK; S.Quenby@warwick.ac.uk; 7 Tommy’s National Centre for Miscarriage Research, University Hospitals Coventry & Warwickshire NHS Trust, Coventry CV2 2DX, UK

**Keywords:** miscarriage, cell-free DNA, cytogenetic analysis, chromosomal abnormalities

## Abstract

Approximately one in four pregnancies result in pregnancy loss, and ~50% of these miscarriages are caused by chromosomal abnormalities. Genetic investigations are recommended after three consecutive miscarriages on products of conception (POC) tissue. Cell-free DNA (cfDNA) has been utilised for prenatal screening, but very little work has been carried out in nonviable pregnancies. We investigated the use of cfDNA from maternal blood to identify chromosomal abnormalities in miscarriage. One hundred and two blood samples from women experiencing a first trimester miscarriage were collected and stored. The mean gestational age was 7.1 weeks (range: 5–11 weeks). In this research, samples without a genetic test result from POC were not analysed. CfDNA was extracted and analysed using a modified commercial genome-wide non-invasive prenatal test. No results were provided to the patient. In 57 samples, cytogenetic results from POC analysis were available. Chromosomal abnormalities were identified in 47% (27/57) of POC analyses, and cfDNA analysis correctly identified 59% (16/27) of these. In total, 75% (43/57) of results were correctly identified. The average cfDNA fetal fraction was 6% (2–19%). In conclusion, cfDNA can be used to detect chromosomal abnormalities in miscarriages where the ‘fetal fraction’ is high enough; however, more studies are required to identify variables that can affect the overall results.

## 1. Introduction

Early pregnancy loss is the most common complication during pregnancy [1], and is defined as miscarriage. One in five pregnancies ends in spontaneous miscarriage [2], and 50% of these are due to chromosomal abnormalities [3]. It is important to identify whether a chromosomal abnormality was the underlying etiology of the pregnancy loss because this may have an indication for the prognosis of future pregnancies. If a sporadic chromosomal abnormality is the cause of the pregnancy loss, the prognosis for future pregnancies is better than if the chromosome complement is normal. In which case, there may be another non-chromosomal, reason for the miscarriage. If there is an unbalanced chromosomal rearrangement in the pregnancy loss, it could mean that one of the parents carries a balanced chromosomal rearrangement. This would mean that future pregnancies would be susceptible to the same or other unbalanced rearrangement. In these cases, it is important to obtain blood samples for parental karyotyping for assessment of recurrence risk.

The Royal College of Obstetricians and Gynaecologists (RCOG) Green-top Guideline No. 17 [4] recommends cytogenetic analysis of pregnancy tissue after the third and subsequent miscarriages, or karyotyping of parental samples if pregnancy tissue is not available. Traditionally, cell culture and G banded chromosomal analysis were used to detect abnormalities in pregnancy tissue. However, there is often a high failure rate, due to the poor quality of tissue received, the difficulty with culturing cells from such tissues and a limited resolution in detecting micro-deletion and duplication syndromes. Therefore, molecular-based approaches, such as quantitative fluorescent PCR (QF-PCR) and microarray have been implemented across laboratories.

Currently, genetic testing for miscarriage is completed on pregnancy tissue, which comprises of placental and fetal components, referred to as products of conception (POC). This tissue needs to be fresh, uncontaminated, and unfixed so that the fetal tissues can be identified and have DNA extraction or cell culture performed. This comes with the risk of potential maternal cell contamination (MCC) which could lead to misdiagnosis of the sample. The POC samples contain maternal tissues intertwined with fetal tissues. Maternal cells can be carried over during the selection of fetal tissues resulting in maternal DNA during DNA extraction or an overgrowth of maternal cells during cell culture. Moreover, in many cases, POC are unavailable, or unreturned by the patient.

Cell-free DNA (cfDNA) was first identified by Dennis Lo [5] who demonstrated that small fragments of cfDNA from the plasma of pregnant women represent the entire fetal genome. Although cfDNA is already utilised for prenatal screening, very little work has been carried out in nonviable pregnancies to date. Only two studies by Clark-Ganheart el al. and Yaron et al. [6,7] have evaluated the use of cfDNA in a miscarriage setting.

A prospective cohort study Clark-Ganheart et al. [6] analysed 50 cfDNA samples of non-viable pregnancies. Gestational age determined by ultrasound scan ranged from 6.1 to 38.4 weeks. Among these, 38 of the 50 samples had a reportable result, including eight samples which demonstrated trisomies. The study by Yaron et al. [7] tested cfDNA to analyse pregnancy loss at less than 14 weeks. In total, 86 pregnancies had cfDNA results with comparable POC (from CVS sampling). The median fetal fraction was 5%. Out of the 86 samples, 55 (64%) had a chromosomal abnormality and 30 of those (55%) were detected using standard non-invasive prenatal testing (NIPT) log-likelihood ratio (LLR) cut-offs. To increase the sensitivity, a pregnancy-loss specific threshold was developed using a 50 sample ‘training set’. This increased the detection rate to 82%.

CfDNA would be extremely useful to ascertain chromosomal causes of miscarriages at the point of miscarriage diagnosis by a simple blood test. This study investigates how cfDNA can be utilised to detect chromosomal abnormalities in miscarriage and to compare the results with those of POC testing.

## 2. Materials and Methods

### 2.1. Ethical Approval

The study was completed at Tommy’s National Centre for Miscarriage Research, with IRAS project ID, 215646, that received Research Ethics Approval (REC reference: 16/WM/0423, 23/11/2016, West Midlands-South Birmingham Research Ethics Committee) and Health Research Authority (HRA) approval.

### 2.2. Patient Samples

Informed consent was obtained from patients experiencing early miscarriage and seen at Tommy’s National Centre for Miscarriage Research hosted by Birmingham Women’s and Children’s Hospital NHS Trust and University Hospital Coventry & Warwickshire NHS Trust between February 2017 and July 2019. The consent explicitly included consent to work with the patient’s POC and genetic material. Samples were collected as donations to medical research and the tissue(s) were handled in accordance with the Human Tissue Act (HTA). The donors maintained their ability to withdraw consent for further use but did not retain any rights to the samples after acquisition.

Eligibility criteria included maternal age over 16 years and a gestational age of <12 weeks confirmed by ultrasound scan at the time of miscarriage diagnosis with pregnancy tissue remaining in situ. Samples were included in analysis in cases where there was a cytogenetic result from corresponding POC analysis, except in the case of seven known triploid cases, which were excluded.

Blood samples were taken for cfDNA analysis and to assess βhCG levels. Up to 10 mL of maternal blood was collected for cfDNA in cell-free DNA BCT (STRECK) tubes, and crown-rump length (CRL) measurements were taken by ultrasound where possible to assess the fetal gestation. Chromosomal abnormalities obtained from POC testing were communicated to the patient via standard patient care. CfDNA results were not shared with the patient. 

### 2.3. Sample Processing 

Plasma was isolated from whole blood using double centrifugation and transferred into a DNA LoBind tubes (Eppendorf) in 1 mL aliquots. These aliquots were stored at −80 °C until use.

### 2.4. Cytogenetic Analysis

Products of conception (POC) were collected as routine clinical samples and targeted QF-PCR and chromosomal microarray analysis (CMA) was completed on POC after the third and subsequent consecutive miscarriages(s) according to the RCOG Green-top Guideline No. 17 [4]. 

QF-PCR trisomy screen was first performed on DNA from POC to test for trisomies 13, 18 or 21, triploidy and sex chromosome aneuploidy. If the QF-PCR was abnormal, then it was reported. If it was normal, then CMA testing was carried out using OGT CytoSure 8 × 60 k Constitutional v3 design; exon/gene level resolution of ~500 DDD/ClinGen curated developmental genes and syndromic regions; tiered backbone resolution ~120–500 kb; analysis in build GRCh37 using CytoSure v4.9 and CBS algorithm. The microarray analysis detected copy number imbalances >1 Mb and in some cases had higher resolution.

### 2.5. Cell-free DNA Testing

Plasma (1 mL) from patients who had consented to testing by an external laboratory were submitted to the Illumina laboratory in Cambridge and processed in a 24-sample batch through a modified Illumina VeriSeq NIPT solution v2 workflow as previously described [8,9], but using the latest analysis platform [10] and with a few small modifications. 

## 3. Results

**Figure 1:**
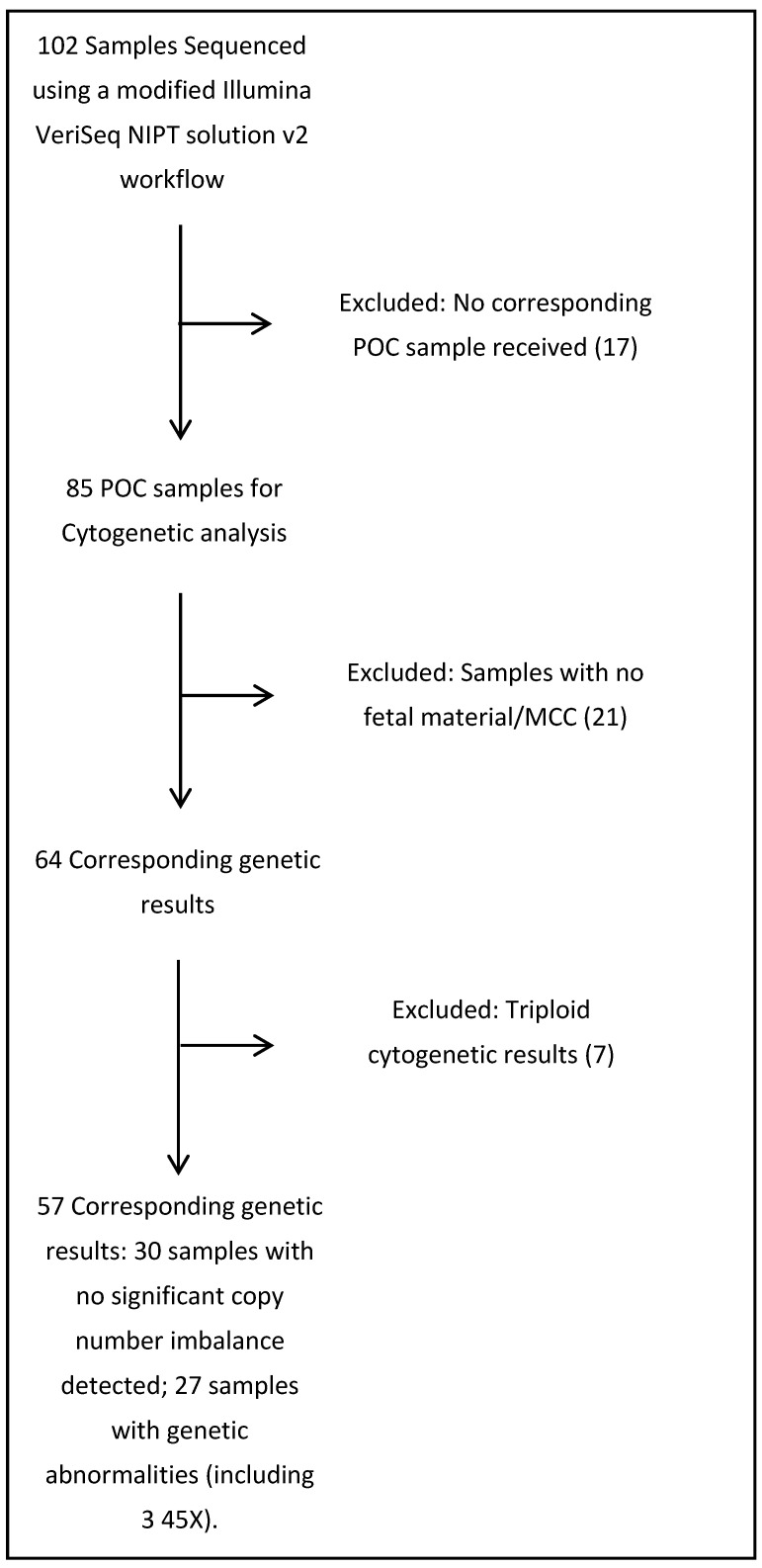
Flow chart describing inclusion/exclusion of cfDNA samples. MCC: maternal cell contamination; NIPT: on-invasive prenatal testing; POC: products of conception.

In total, 102 cfDNA samples were collected once a miscarriage had been confirmed. All 102 samples were analysed by VeriSeq NIPT v2 Solution analysis on a NextSeq500. Eighty-five corresponding POC samples were received. In total, 64 pregnancies had a corresponding cytogenetic result from POC analysis, 21 POC samples were not suitable for analysis and 17 cfDNA samples did not receive corresponding POC samples. The 17 unreceived POC samples and the 21 POC samples not suitable for analysis were excluded from cfDNA analysis, and known triploid pregnancies were excluded (Figure 1). 

Chromosomal abnormalities were identified by POC analysis in 27/57 (47%) cases. Patient baseline characteristics are summarised in Table 1. From the 57 samples with corresponding cytogenetic analysis, the average age was 34 years (20–43 years), with a clinical gestation of 7.1 weeks (5–11 weeks) and a fetal fraction of 6% (2–19%). In total, 70% (40/57) of samples including 16/27 (59%) of genetic abnormalities and 27/30 (90%) of genetically normal samples were identified correctly using VeriSeq. This corresponds to a sensitivity of 59% (16/27), specificity of 90% (27/30) and accuracy of 75% (43/57), although the sample cohort was relatively small.

CfDNA analysis correctly identified 43/57 (75%) of samples including 16 abnormal and 27 normal samples. Table 2 compares POC results and cfDNA test results of the cases where an abnormal result was detected in POC. The following anomalies were detected in POC: common trisomies (3), monosomy X (2), common trisomy combined with 45, X (1), monosomy 21 (1), full rare trisomies (14), mosaic rare trisomies (2) and copy number variations (4). Amongst the rare trisomies, trisomy 22 and trisomy 15 were the most frequent. Fetal fractions were from 3–12% (mean 5%). CfDNA results were fully concordant with POC results in 40/57 samples. CfDNA results generated normal results in 27/30 cases, and discrepant results in 3/30 cases of known normal cases. The two mosaic samples (sample IDs 51 and 586) were not correctly identified with cfDNA testing; however, other imbalances were detected in those samples. Sample ID 228 gave a monosomy 21 result on POC, but cfDNA testing identified CNVs in several other chromosomes. 

Four of the samples were from miscarriages where subchromosomal deletions and duplications were identified by POC analysis (sample IDs 133, 202, 303 and 319). A 56 Mb duplication at 7q22.1q36.3 and a 21 Mb terminal duplication at 19q13.12q13.43 were detected by cfDNA analysis. A 70 Mb deletion at 13q13.3q34, a 6 Mb terminal deletion at 7q36.2q36.3, a 9 Mb terminal duplication at 4q34.3q35.2 and a 30 Mb terminal deletion at 5q33.1q35.3 were not detected by cfDNA analysis. 

The results were grouped into three categories using different gestations, βhCG values and fetal fraction cut offs, to see if this could improve the result calling between cfDNA and POC cytogenetic analysis (Table 3). The gestation was split into four groups, <7 weeks, 7–8 weeks, ≥8 and unknown gestation. As the gestation increased in these groups, the correctly identified chromosomal abnormalities from cfDNA testing increased. The βhCG value was split into three groups of <8000, 8000–35,000 and >35,000 mIU/mL. As the βhCG value increased in these groups, the correctly identified chromosomal abnormalities from cfDNA testing also increased. The fetal fraction groups were split into three groups: <5, 5–8 and ≥9. Again, as fetal fraction groups increased so did the percentage of correctly identified chromosomal abnormalities from cfDNA testing. 

## 4. Discussions

Our cfDNA study cohort was recruited through Tommy’s National Centre for Miscarriage Research, at Birmingham Women’s Hospital and University Hospital Coventry and Warwickshire. In total, 102 samples were evaluated using modified VeriSeq NIPT V2 (Illumina), and 57 samples were analysed with corresponding POC cytogenetic analysis. 

The cfDNA analysis was separated into three categories for analysis (Table 3). Whilst some chromosomal abnormalities were identified at lower fetal fraction, at <5% fetal fraction, only 60% of samples were correctly identified, and of those, most were from euploid pregnancies. In contrast, where the fetal fraction was ≥9%, 100% of cytogenetic results were correctly identified. In our study, we note that the majority of abnormalities can be detected above 5% fetal fraction. However, it is difficult to define an exact cut off due to the low sample numbers and biological variation. 

The discrepancies we observed between the POC genetic results and the cfDNA testing could be caused by confined placental mosaicism. CfDNA analysis tests DNA derived from the placenta/cytotrophoblasts only, whereas the POC testing may consist of fetal tissue and whole placental tissue. This could result in a discrepancy between the results. In two cases, mosaic genetic abnormalities were identified in the POC analysis which cfDNA testing did not identify. These results could be due to confined placental mosaicism for the trisomic cells or due to the current limitation of the method. Mosaicism is difficult to diagnose with any methodology, and it is possible that cfDNA analysis could become a helpful adjunct to current POC testing in detecting biologically relevant abnormal cell lines.

**Figure 2:**
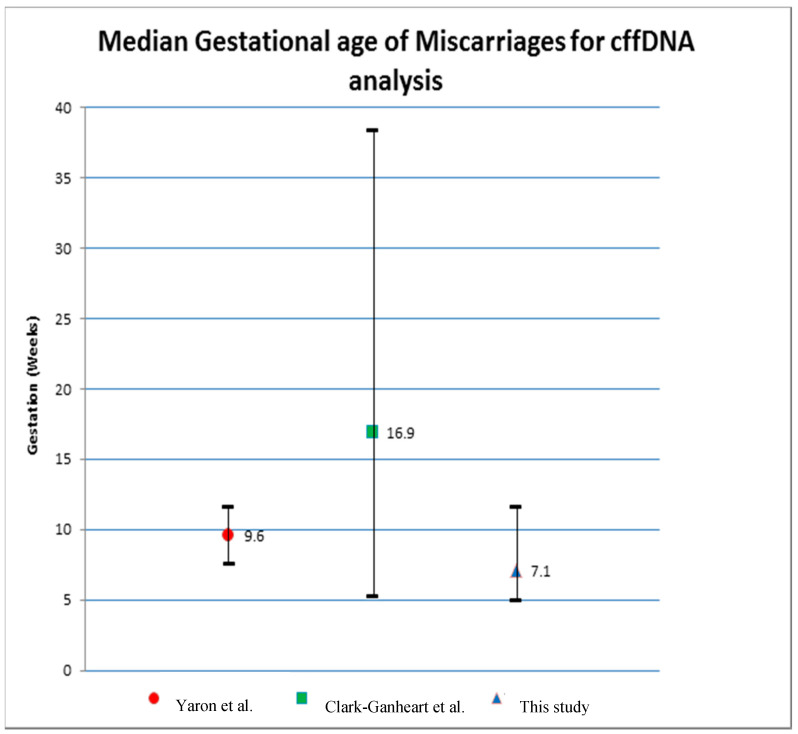
Gestations of pregnancy loss by ultrasound scan [6,7].

Tommy’s National Centre for Miscarriage Research is specialised in the care of families undergoing recurrent miscarriage. These families are very aware of when they first become pregnant and benefit from careful monitoring during their first trimester. Consequently, the miscarriages in our study cohort were diagnosed earlier than in other studies. Clark-Ganheart et al. [6] recorded gestational ages of 16.9 (6.1–37.2) weeks, and Yaron et al. [7] recorded gestational ages 9.6 (5.1–13.6) weeks (Figure 2). 

This study and others have demonstrated that in the majority of cases of pregnancy loss where the pregnancy tissue is still in situ, it is possible to detect chromosomal abnormalities using cfDNA. This study correctly identified 59% of chromosomal abnormalities with a 75% concordance to POC results. In comparison, Clark-Ganheart et al. [6] had 87.5% concordant results where there was an available cytogenetic result, and Yaron et al. [7] had 82% concordant results using pregnancy loss-specific LLR thresholds. Using 50 cases as a training set, Yaron et al. [7] established a pregnancy loss-specific LLR threshold. Overall detection was 82% on 86 non-mosaic cases. This was achieved after identifying a pregnancy-loss-specific LLR based on a training set. This indicates that the LLR needed for this cohort may need to be different from singleton pregnancies. In comparison, our study used the standard NIPT LLR cut-offs to analyse cfDNA, and it is feasible that having a pregnancy-loss-specific LLR would improve the detection rate. The next step in this study may be to conduct a trial using an algorithm similar to the one proposed by Yaron et al. [7], using a pipeline with modified LLRs to optimise the detection rate of all autosomal trisomies for this cohort of patients.

This study and others [6,7] have shown that cfDNA can be utilised to assess the genetic contribution to miscarriage. However, there are still some genetic abnormalities that can be missed, dependent on the assay used (e.g., triploid and mosaic samples, and autosomal trisomies at low fetal fraction/low gestation). Triploid cases were excluded in both this study and Yaron et al. [7] as they were not detectable by the modified Illumina VeriSeq NIPT solution v2 workflow. However, a single nucleotide polymorphism-based platform for analysis of cfDNA should be able to identify triploid cases.

CfDNA cannot completely replace current cytogenetic testing. A recurrent pregnancy loss algorithm was proposed by Yaron et al. [7] which would utilise cfDNA testing in recurrent pregnancy loss. 

When a third or subsequent pregnancy loss has been diagnosed, current guidelines by the Royal College of Obstetricians and Gynaecologists (RCOG) Green-top Guidelines No. 17 [4] are to test the pregnancy tissue for fetal aneuploidies. Alongside this routine testing, a maternal blood sample could be collected to complete cfDNA testing. If an aneuploidy is detected in cfDNA testing and explains the reason for the miscarriage, no further work is required as numerical errors usually occur sporadically and the likelihood of a successful subsequent pregnancy is not negatively affected. As cfDNA only detects an unbalanced chromosomal abnormality, if no chromosomal abnormality is identified using cfDNA testing, then cytogenetic analysis on POC should be recommended to see if there is a chromosomal abnormality that is not detectable by cfDNA testing (e.g., CNVs, triploid or mosaic samples). This would reduce the number of POC tests required and could achieve a result for more patients where there is no POC available. It is important to note that some chromosomal abnormalities would still be missed if POC is not available. In cases where an unbalanced rearrangement is identified that could be due to an inherited or de novo Robertsonian or reciprocal translocation, parental karyotyping should be recommended to assess whether one (or both) of the parents is a carrier of this translocation.

## 5. Conclusions

Knowing the genetic result of a pregnancy loss can be applied during counselling patients for the prognosis of future pregnancies. It may also be helpful to provide psychological support and relief from the guilt that can be associated with pregnancy loss. 

Using cfDNA to identify whether a miscarriage was caused by chromosomal abnormalities would have a huge clinical impact upon patients for whom conventional cytogenetic testing may not be available, either due to the unavailability of pregnancy tissue for testing or patient preferences. However, cfDNA testing is only feasible where the pregnancy remains in situ at the time of miscarriage diagnosis. 

We have demonstrated that in some cases, cfDNA can be used to detect a genetic aberration in miscarriages providing the maternal plasma sample is collected when the pregnancy tissue is still in situ and in cases where there is enough fetal fraction. Further work is required to improve this testing and to identify variables that can affect the overall results so that it may be applied clinically.

## Figures and Tables

**Table 1:** Characteristics of cfDNA samples with corresponding products of conception (POC) results suitable for analysis (excluding triploid pregnancies).

	Total (*n* = 57)	Chromosomally Normal (*n* = 30)	Chromosomally Abnormal (*n* = 27)
Maternal age (years) (mean and range)	34 (20–43)	31 (20–41)	37 (24–43)
Previous losses (mean and range)	3.8 (0–14)	4.1 (0–14)	3.3 (0–6)
Gestational age (weeks) (mean and range)	7.1 (5–11)	7.4 (5–11)	6.4 (5–9.3)
βhCG (mIU/mL) (mean and range)	38,356 (69–263,766)	50,632 (69–263,766)	21,538 (491–100,638)
Fetal fraction (%) (mean and range)	6 (2–19)	7 (2–19)	5 (3–12)

**Table 2:** Analysis of cfDNA using a modified Illumina VeriSeq non-invasive prenatal testing (NIPT) solution v2 workflow compared to the genetic outcomes of microarray analysis of positive POC results.

Sample ID	Gestation (Weeks)	CRL (mm)	BhCG (mIU/mL)	Maternal Age (Years)	No. of Previous Losses	POC Results	POC Sex	CfDNA Results	CfDNA Sex	Fetal Fraction
4	7 + 2	2.8	19,247	40	2	Trisomy 22	Female	NO ANOMALY DETECTED	Female	4%
51	5 + 4	2	12,725	34	6	Mosaic trisomy 4	Male	DETECTED: del (10) (p15.3q21.1)	Male	4%
99	6 + 0	not recorded	5111	27	4	Trisomy 5	Male	NO ANOMALY DETECTED	Male	6%
133	not recorded	not recorded	57,348	34	4	Terminal deletion at 7q36.2q36.3 (6 Mb) and terminal duplication at 19q13.12q13.43 (21 Mb)	Female	DETECTED: dup (19) (q13.12q13.43)	Female	7%
163	8 + 4	20.1	13,466	24	3	Turners, 45 X	Female	DETECTED: XO	Female	7%
164	6 + 0	9	6774	42	2	Trisomy 15	Female	DETECTED: +15	Female	5%
175	8 + 0	No FP seen	5323	34	6	Turners, 45 X	Female	DETECTED: XO	Female	5%
176	7 + 0	14	491	43	2	Trisomy 15	Female	DETECTED: +15	Female	5%
202	6 + 0	5	13,819	29	4	Terminal duplication at 4q34.3q35.2 (9 Mb) and terminal deletion at 5q33.1q35.3 (30 Mb)	Female	NO ANOMALY DETECTED	Female	4%
228	7 + 0	12	6220	41	5	Monosomy 21	Male	DETECTED: dup (15) (q21.3q23); dup (20) (q11.21q13.12)	Male	3%
245	not recorded	6.5	14,762	33	3	Trisomy 22	Male	NO ANOMALY DETECTED	Male	4%
260	7 + 0	not recorded	5194	43	2	Trisomy 22	Male	NO ANOMALY DETECTED	Male	4%
264	6 + 0	4	14,002	33	Not recorded	Trisomy 13.	Female	DETECTED: +13; +16	Female	4%
279	6 + 0	6	8429	40	4	Trisomy 7	Female	DETECTED: +7	Female	12%
287	not recorded	4	not recorded	42	0	Trisomy 12	Female	DETECTED: +12	Female	4%
290	5 + 0	5.2	44,313	39	0	Trisomy 16	Female	DETECTED: +16	Female	7%
303	6 + 0	1.9	34,087	35	3	Deletion at 13q13.3q34 (70 Mb)	Female	NO ANOMALY DETECTED	Female	5%
319	7 + 0	9.3	13,642	40	6	Duplication 7q22.1q36.3 (56 Mb)	Female	DETECTED: dup (7) (q22.1q31.1)	Female	7%
328	not recorded	4.4	2983	42	3	Trisomy 11	Female	NO ANOMALY DETECTED	Female	4%
400	5 + 6	4.6	29,052	42	5	Trisomy 22	Male	DETECTED: +22	Male	3%
462	6 + 1	3	22,429	40	2	Trisomy 15	Male	DETECTED: +15	Male	5%
519	9 + 3	26.31	100,638	28	6	Trisomy 21	Male	DETECTED: +21	Male	10%
529	7 + 0	not recorded	21,171	39	5	Trisomy 21 and monosomy X.	Female	DETECTED: +21	Female	5%
541	7 + 3	12.02	42,333	33	2	Trisomy 15	Female	DETECTED: +15	Female	8%
586	6 + 0	5.28	22,435	41	2	Mosaic trisomy 17	Female	DETECTED: del (6) (p25.1p22.3); +18	Female	4%
816	5 + 0	no FP seen	5852	40	3	Trisomy 18.	Female	NO ANOMALY DETECTED	Female	4%
965	7 + 6	14.7	73,962	42	3	Trisomy 15	Male	DETECTED: +15	Male	INVALIDATED

CfDNA results labelled in green are concordant with POC results and results labelled in red are discordant with POC results.

**Table 3:** CfDNA vs karyotype of POC.

		CfDNA Results	
		Correctly Identified (%)	Not Identified (%)
	Total	43 (75.4)	14 (324.6)
**Gestation (weeks)**	<7	14 (66.7)	7 (33.3)
	7–8	10 (76.9)	3 (23.1)
	≥8	11 (100.0)	0 (0.0)
	Unknown	8 (53.3)	7 (46.7)
**βhCG** **(mIU/mL)**	<8000	9 (60.0)	6 (40.0)
	8000–35,000	14 (66.7)	7 (33.3)
	>35,000	19 (95.0)	1 (5.0)
**Fetal Fraction (%)**	<5	13 (59.1)	9 (40.9)
	5–8	19 (79.2)	5 (20.8)
	≥9	10 (100.0)	0 (0.0)
